# Sea cucumbers bioturbation potential outcomes on marine benthic trophic status under different temperature regimes

**DOI:** 10.1038/s41598-023-38543-6

**Published:** 2023-07-18

**Authors:** Claudia Ennas, Viviana Pasquini, Hiba Abyaba, Pierantonio Addis, Gianluca Sarà, Antonio Pusceddu

**Affiliations:** 1 Dipartimento di Scienze Della Vita e Dell’Ambiente, Università Degli Studi Di Cagliari, 09126 Cagliari, Italy; 2 Scuola Universitaria Superiore IUSS Pavia, 27100 Pavia, Italy; 3 Dipartimento di Scienze Della Terra e del Mare, Università Degli Studi di Palermo, 90123 Palermo, Italy

**Keywords:** Marine biology, Carbon cycle

## Abstract

Eutrophication affects coastal oceans worldwide, modifies primary production and sediment biogeochemistry and, overall, is progressively compromising marine ecosystems’ integrity. Because of their known bioturbation ability, sea cucumbers are supposed to be candidates for mitigating benthic eutrophication. To provide insights on this, we investigated differences in organic matter quantity and biochemical composition (as proxies of benthic trophic status) of sediments and feces of the sea cucumber *Holothuria tubulosa* acclimated in mesocosms at temperatures comprised between natural conditions (14–26 °C) and an extreme of 29 °C (representing the highest anomaly under heat waves in the Mediterrranean Sea). Organic matter features differed significantly between sediments characterized by different trophic statuses and the holothuroid’s feces, though with some exceptions. Feces resulted almost always organically enriched when compared with the ambient sediments, though with variable differences in composition in sediments characterized by different initial trophic status. Our results point out that sea cucumbers maintain their bioreactor capacity at all experimental temperatures including the (anomalous) highest one, irrespectively of the available food, suggesting that they could be profitably utilized to mitigate benthic eutrophication also in a warmer Mediterranean Sea.

## Introduction

Eutrophication is a typology of exacerbated anthropogenic disturbance which occurs locally in worldwide marine coastal ecosystems where the nutrients excess derives from a variety of anthropogenic activities^1–4^. The main sources of eutrophication include coastal urbanization, agrozootechnical activities, aquaculture, industrialization, tourism development, and lack or malfunction of wastewaters treatment systems^5–8^. Nowadays, eutrophication represents one of the greatest stressors for coastal marine ecosystems worldwide, contributing to increased frequency, duration, and extent of algal blooms, and also affecting sediment biogeochemistry and benthic micro-, meio-, and macrofauna communities^5,9–11^, ultimately worsen because of climate change^12^.

In the last decades, several European seas have become progressively more prone to coastal eutrophication^13,14^, and this applies particularly to the Mediterreanean Sea^15^, a semi-enclosed miniature ocean^16^, where the effects of eutrophication could therefore be exacerbated. Along with management plans put in place to limit the nutrient inputs into the seas^13,14^, bioremediation and biomanipulation actions could represent useful tools to reduce or counteract the effects of eutrophication on marine sediments^17,18^.

In this context, deposit-feeding sea cucumbers, important components of the marine benthic biodiversity, are able, thanks to their feeding behavior, to intercept and transform surplus organic matter derived from human activities such as aquaculture^19,20^. They are among the most effective seafloor bioturbators, and their digestive system can be considered a real bioreactor where nutrients from ingested organic matter can be quickly assimilated^19,21–24^. Also, these animals can grow faster in mariculture-impacted sites where protein-enriched feed is abundant^25,26^. They, indeed, have recently been tested and used as bioremediators in polycultures and Integrated Multi-Trophic Aquaculture (IMTA) systems, with promising results^20,27–31^. Despite warm temperatures may negatively affect their metabolic machinery and other functional traits such as, for example, those traits involved in the immune response^32–34^, sea cucumbers are ectothermic and osmo-conformers^35^. Besides this, some holothuroids (i.e., *Holothuria scabra*) have shown tolerance and adaptability to thermal stress after an initial disturbance in energy balance due to the increase in temperature^36,37^, as well as to other environmental stressors such as chronic salinity fluctuations^38,39^.

The Mediterranean sea cucumber *Holothuria tubulosa* (Gmelin, 1788) in particular, is among the most active deposit-feeders able to modify sedimentary organic features^40–46^. This species can tolerate a wide array of physicochemical stressors^42^, and juveniles easily survive under controlled thermal conditions, up to at least 30°C^47^. Their elevated functional plasticity makes them ideal candidates for benthic remediation^19^ under increasing organic enrichment due to the eutrophication or direct influence of human activities under different temperature regimes.

Here, with the aim to improve our understanding of the effectiveness of using sea cucumbers as bioreactors to mitigate benthic eutrophication under different temperature regimes, we designed an experiment to test the null hypothesis by which, under different trophic status conditions, sedimentary organic matter content and biochemical composition (expressed as protein, carbohydrate and lipid concentrations), here used as proxies of benthic trophic status^48^, should not vary between ambient sediments and feces of *H. tubulosa* specimens acclimated under different temperatures.

## Results

### Quantity and biochemical composition of sediment and holothuroid feces at different temperatures

Protein, carbohydrate, lipid, and biopolymeric C (BPC) contents of sediment and feces are provided in Supplementary Table 1. Sedimentary contents of all classes of organic compounds were characterized by a significant effect of the Matrix × Site × Temperature interaction (Table 1).

**Table 1:** Results of the PERMANOVA tests carried out to investigate differences in the quantity and biochemical composition of organic matter between the two matrices (M; sediments vs. feces) subjected to different temperatures (T; 14, 17, 20, 23, 26, 29 °C) in the two sites (S; meso-eutrophic vs. oligo-mesotrophic).

Variable	Source	df	MS	Pseudo-F	P(MC)	% EV
Protein	Matrix (M)	1	6.814	39.389	**	6.2
	Site (S)	1	15.809	91.381	**	14.5
	Temperature (T)	5	2.606	15.064	**	6.8
	M × S	1	5.399	31.208	**	9.7
	S × T	5	2.651	15.324	**	13.8
	M × T	5	2.740	15.840	**	14.3
	M × S × T	5	2.756	15.930	**	28.8
	Residual	48	0.173			5.8
Carbohydrate	Matrix (M)	1	13.526	80.224	**	18.1
	Site (S)	1	24.514	145.400	**	33.0
	Temperature (T)	5	0.373	2.214	ns	0.8
	M × S	1	9.660	57.294	**	25.8
	S × T	5	0.259	1.536	ns	0.7
	M × T	5	0.843	4.997	**	5.5
	M × S × T	5	0.647	3.840	**	7.8
	Residual	48	0.169			8.2
Lipid	Matrix (M)	1	7.448	33.924	**	6.6
	Site (S)	1	10.878	49.547	**	9.7
	Temperature (T)	5	2.726	12.415	**	6.8
	M × S	1	4.968	22.630	**	8.6
	S × T	5	2.697	12.285	**	13.5
	M × T	5	3.210	14.621	**	16.3
	M × S × T	5	3.079	14.026	**	31.2
	Residual	48	0.220			7.2
Biopolymeric C	Matrix (M)	1	10.641	64.988	**	10.1
	Site S)	1	21.458	131.040	**	20.6
	Temperature (T)	5	1.882	11.493	**	5.0
	M × S	1	7.961	48.619	**	15.1
	S × T	5	1.857	11.342	**	9.8
	M × T	5	2.129	13.000	**	11.4
	M × S × T	5	2.073	12.658	**	22.2
	Residual	48	0.164			5.7
Biochemical composition	Matrix (M)	1	27.788	49.520	**	9.4
	Site (S)	1	51.201	91.244	**	17.4
	Temperature (T)	5	5.705	10.167	**	5.3
	M × S	1	20.027	35.690	**	13.4
	S × T	5	5.607	9.993	**	10.4
	M × T	5	6.793	12.105	**	12.8
	M × S × T	5	6.483	11.552	**	24.4
	Residual	48	0.561			6.9

*df* degrees of freedom, *MS* mean square, *Pseudo-F* F statistic, *P (MC)* probability level after Monte Carlo simulations (**p < 0.01; *p < 0.05; *ns* not significant), *% EV* percentage of explained variance.

**Figure 1:**
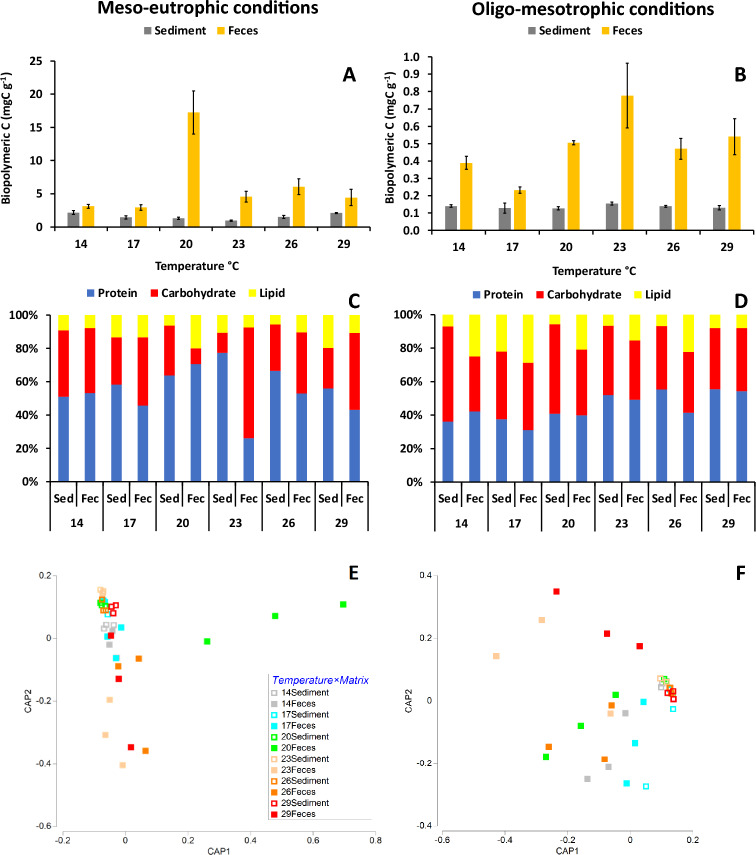
Changes in biopolymeric C (BPC) contents (**A**,**B**) and protein, carbohydrate, and lipid percentage contributions to BPC (**C**,**D**) in sediment (Sed) and feces (Fec) at the six acclimation temperatures (14, 17, 20, 23, 26, 29 °C) in meso-eutrophic (left panel) and oligo-mesotrophic (right panel) conditions. The error bars indicate the standard errors (n = 3). Reported are also the biplots obtained after CAP analysis illustrating differences in biochemical composition of organic matter in sediments and feces separately for meso-eutrophic (**E**) and oligo-mesotrophic (**F**) conditions.

In meso-eutrophic conditions feces were from 2 to 13 times significantly richer in BPC than the relative ambient sediment at all temperatures, except at 14 and 29 °C (Table 2; Fig. 1A). Protein and lipid contents were significantly higher in feces than in the sediment only at 20 °C (ca. 15 times for proteins, 58 times for lipids) and 26 °C (3 times for proteins, 8 times for lipids) (Table 2; Supplementary Fig. 1A–C). Feces carbohydrate content was significantly higher than that in the sediment at all temperatures (from 3 to 26 times), except at 14 and 29 °C (Table 2, Supplementary Fig. 1B). Under meso-eutrophic conditions, the biochemical composition of organic matter differed significantly between feces and meso-eutrophic sediments at all temperatures, except at 14 and 29 °C (Fig. 1B). Such differences varied across temperatures. More in details, at 17, 23 and 26 °C feces were characterized by protein (by 20–77%) and carbohydrate (by 36–66%) contributions to BPC lower and higher, respectively, than in the sediment. At 20 °C feces, when compared to the corresponding sediment, were characterized by higher protein (64 and 71% in sediment and feces, respectively), higher lipid (6 and 20%) and lower carbohydrate (30 and 9%) contributions to BPC (Fig. 1B). At the lowest temperatures (14 and 17 °C) feces and sediments were characterized by relatively similar biochemical composition, whereas the largest differences occurred at 20 °C. At 23–29 °C differences persisted but appeared relatively less marked than those at 20 °C (Fig. 1C).

**Table 2:** Results of the pairwise tests carried out to assess differences in contents of protein, carbohydrate, lipid, biopolymeric C, and biochemical composition between matrixes (sediments vs. feces) in meso-eutrophic and oligo-mesotrophic conditions, at six temperatures (14, 17, 20, 23, 26, 29 °C).

Variable	Temperature °C	t	P(MC)	t	P(MC)
		Meso-Eutrophic		Oligotrophic	
Protein	14	1.862	ns	4.099	*
	17	1.135	ns	0.764	ns
	20	4.614	*	2.741	*
	23	1.217	ns	2.603	ns
	26	4.195	*	11.389	***
	29	1.401	ns	6.745	**
Carbohydrate	14	2.409	ns	4.28	*
	17	3.632	*	7.28	**
	20	19.215	***	2.828	*
	23	7.308	**	2.773	ns
	26	2.975	*	2.874	*
	29	2.371	ns	2.705	ns
Lipid	14	0.668	ns	4.795	*
	17	2.48	ns	1.194	ns
	20	4.205	*	4.857	**
	23	2.744	ns	4.778	**
	26	3.158	*	4.705	*
	29	0.431	ns	2.665	*
Biopolymeric C	14	2.243	ns	6.41	**
	17	3.086	*	3.057	*
	20	4.893	**	24.477	**
	23	4.306	*	3.333	*
	26	3.766	*	5.495	**
	29	1.908	ns	3.929	*
Biochemical composition	14	2.069	ns	4.641	**
	17	2.746	*	1.636	ns
	20	4.446	*	3.416	**
	23	6.538	**	3.006	*
	26	3.12	*	3.63	**
	29	2.243	ns	2.946	*

t = statistic t; p(MC) = probability level after Monte Carlo simulation; *p < 0.05; **p < 0.01; ***p < 0.001; *ns* not significant.

In oligo-mesotrophic conditions, feces were from 2 to 5 times significantly richer in BPC than the relative ambient sediments at all temperatures (Fig. 1D). Feces protein content was from 2 to 4 times significantly higher than that in the sediment at all temperatures, except at 17 and 23 °C (Table 2; Supplementary Fig. 1D). Feces carbohydrate content was 2–3 times significantly higher than that in the sediment at all temperatures, except 23 and 29 °C (Table 2; Supplementary Fig. 1E). Feces lipid content was from 4 to 15 times significantly higher than that in the sediment at all temperatures, except at 17 °C (Table 2; Supplementary Fig. 1F). Under oligo-mesotrophic conditions, the organic matter biochemical composition differed significantly between feces and the corresponding sediment at all temperatures, except at 17 °C (Table 2). Such differences were due to lipid contributions to BPC in feces generally higher than those in the corresponding sediment at all temperatures, but at 29 °C, accompanied by lower carbohydrate contributions to BPC at 14 and 20 °C (Fig. 1E). Differences in the organic matter biochemical composition between feces and the corresponding sediment persisted at all temperatures, with differences at the lowest temperatures (14 and 17 °C) less marked than those at the higher ones (20–29 °C) (Fig. 1F).

### ***Magnitude of feces organic enrichment***

**Figure 2:**
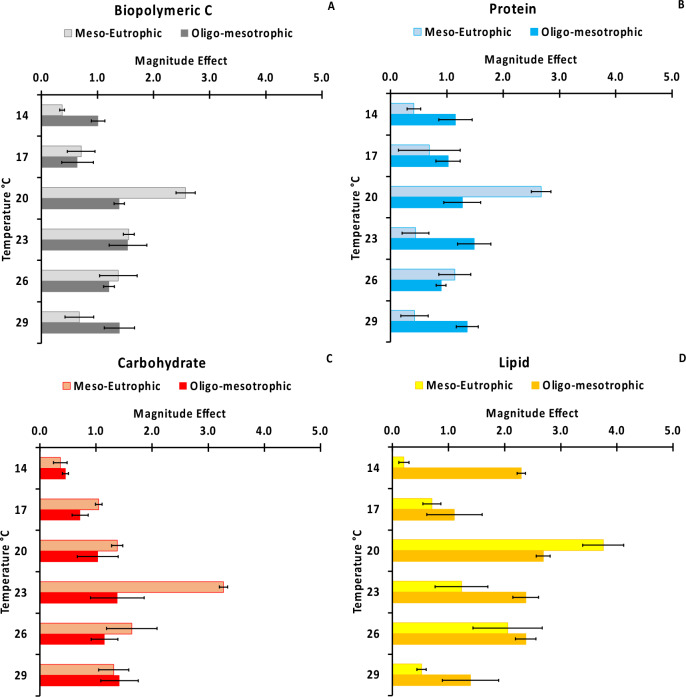
Magnitude of the effects of different temperatures on the biopolymeric C (**A**), protein (**B**), carbohydrate (**C**) and lipid (**D**) enrichment of holothuroid feces in meso-eutrophic and oligo-mesotrophic conditions. The error bars indicate the standard error (n = 3).

Under meso-eutrophic conditions, BPC enrichment of holothuroid feces showed a quasi-unimodal distribution with a snap at 20 °C, whereas under oligo-mesotrophic conditions below 20 °C it was ca. 1.7 times higher than that at lower temperatures (14–17 °C) (Fig. 2A). BPC enrichment of feces in oligo-mesotrophic conditions was higher than that in meso-eutrophic conditions at 14 and 29 °C, lower at 20 °C, and similar at all other temperatures (Fig. 2A).

Under meso-eutrophic conditions protein, lipid and carbohydrate enrichment showed quasi-unimodal distributions across treatments, with the highest values at 20 °C for protein and lipid, and at 23 °C for carbohydrate (Fig. 2B–D). Protein and lipid enrichment of feces in oligo-mesotrophic conditions was higher than that in meso-eutrophic ones at 14, 23, and 29 °C, while the opposite was observed at 20 °C. Carbohydrate enrichment in oligo-mesotrophic conditions was generally lower than or equal to that in meso-eutrophic conditions at all temperatures. Under oligo-mesotrophic conditions protein enrichment of feces did not vary among treatments, the lipid one was lowest at 17 and 29 °C, and the carbohydrate one slightly increased with temperature, resulting at 29 °C ca. 3 times higher than that at 14 °C (Fig. 2B–D).

## Discussion

Eutrophication is among the most recurrent phenomena in coastal oceans worldwide^1–3^. Eutrophication, mostly caused by increased inorganic nutrient inputs in the seawater generated by urban, inland agriculture and industrial wastes, can also determine the accumulation, over sustainable thresholds, of organic matter produced by activities at sea^48^, like mariculture^5,49^. Integrated multi-trophic aquaculture (IMTA), by potentially transforming mariculture wastes (e.g., uneaten food and fish feces) into food sources for other reared species^27,50–53^, could thus also help to mitigate the impacts of marine aquaculture on the benthic trophic status. In this regard, holothuroids feeding on fish farm waste in integrated aquaculture, are potentially able to modify quantity and composition of sedimentary organic matter^31^. Therfore, they could represent a reliable tool to modify benthic trophic status^19^.

Temperature can affect the nutritional physiology of marine organisms^54–59^, including the holothuroid *H. tubulosa*, one of the most common sea cucumbers of the shallow Mediterranean Sea^46,60^. Based on this assumption, we investigated the potential capacity of *H. tubulosa* to influence sedimentary organic matter pools under different temperatures.

At all temperatures and in both trophic conditions (in terms of biopolymeric C loads), the feces produced by the acclimated *H. tubulosa* specimens were characterized by organic matter contents significantly higher than those of the corresponding sediments. Such enrichment is a well-known feature of this and other holothuroids. For example, Amon et al.^41^ and Mercier et al.^61^ reported that holothuroids, under natural temperature regimes, produce C- and N-enriched feces. Such organic enrichment of feces is, most likely, the result of organic matter concentration in the initial digestive tract (i.e., the esophagus)^19^ and of the selection of food particles from the original sediment (e.g., by chemo-selection^46,62–64^). Organic enrichment of holothuroids feces can also be due to the compression and packing of feces within an organic mucus before excretion^61,65^. Moreover, during the feces transit in the intestine, the ingested sediment is mixed with the digestive fluids and the bacterial flora, which further enriches feces with additional organic C pools^19,40,66–69^.

The magnitude of biopolymeric C enrichment of holothuroids’ feces varies between trophic conditions and across temperatures. Nevertheless, the general organic enrichment of holothuroids’ feces, irrespectively of trophic conditions and temperature, lead us to conclude, unexpectedly, that this species could act as a sort of flywheel of organic matter accumulation and, thus, of benthic eutrophication. Nonetheless, the overall trophic status of incoherent sediments is determined not only by the whole organic loads but also by their biochemical composition (nutritional quality)^48^. In this regard, previous studies reported that deep-sea holothuroids, preferably ingesting large quantities of labile organic material, can influence the overall trophic conditions of deep-sea sediments^22,70^. Based on the richer protein and lipid contents of holothuroids feces, since proteins are more rapidly digested than carbohydrates and lipids are energy-rich compounds^71^, our results suggest that *H. tubulosa* can also enhance sedimentary organic matter nutritional quality. In this sense, holothuroids feces would represent a “fresh” source of organic matter available for benthic consumers and, as such, could foster and accelerate the energy transfer to higher trophic levels: this would partially help to mitigate organic C accumulation due to the sole release of feces. Overall, these results let us conclude that the eventual use of holothuroids to condition the benthic trophic status, whatever the temperature regime, should be accurately calibrated according to the initial trophic status of the sediments, with attention not only to the bulk of organic C but also to its nutritional quality for deposit-feeders. Our results pinpoint also that the role of holothuroids in influencing the organic loads of marine coastal sediments is not profoundly modified under extremely high temperatures (26–29 °C), like those possibly occurring under future marine heat waves. This result suggests that their potential capacity as bioreactors could be similar also under warmer temperature regimes.

Nevertheless, we show here also that the organic enrichment of feces under meso-eutrophic conditions resulted larger than that under oligo-mesotrophic conditions only at 20 °C. At all other temperatures, especially at the warmest ones (26–29 °C), the enrichment resulted equal to or lower than that under oligo-mesotrophic conditions. Such discrepancy is not easily explicable, but could be related with the seasonal variations in holothuroids’ metabolism. The range of temperatures used in our experiment with acclimated specimens also includes temperatures that, with exception of 29 °C, *H. tubulosa* normally faces in the Mediterranean Sea during seasonal transitions. Although *H. tubulosa* is a continuous deposit-feeder^46,72^, an increase in temperature dictated by seasonality can induce a variation of its feeding activity, metabolism and reproduction, and, consequently, of the organic content and biochemical composition of its feces^19,36,40,41,73^. While during the transition from winter to spring, concurrently to the development of the gonads, holothuroids increase their metabolism, later in summer, when spawning occurs^74^, and in fall-winter seasons their metabolism decreases^73,75,76^. During spring (at temperatures close to 20 °C in the shallow Mediterranean Sea^77^), *H. tubulosa* shows a typical gonads’ growing phase, during which it starts eating more food to store energy for the subsequent reproduction period^74^. At 20 °C, holothuroids’ feces were more organically enriched when feeding on meso-eutrophic sediments (richer in organic content) than on poorer oligo-mesoeutrophic sediments and that most of the organic excess under the meso-eutrophic conditions was due to increased contributions of relatively more labile molecules (i.e., protein and lipids^71^). This result would thus suggest that the spring phase of gonads’ growth, characterized by the release of more organically enriched feces, could vary with bioavailable sedimentary C loads. In meso-eutrophic sediments, the whole amount of organic C available for holothuroids could exceed the amount of storable substrates, whereas in oligo-mesotrophic conditions, the lower amount of available organic loads could be more conservatively assimilated by the animals, thus leading holothuroids to produce less organically enriched feces. This hypothesis, however, must be considered with caution and, further, tested experimentally. Whatever the physiological trigger of the holothuroids’ metabolism, our results suggest that their use in the conditioning of the benthic trophic status should be accurately calibrated also to the season of the year and, thus, to their life stage.

The results of our study are not fully explicative of the mechanistic processes behind the observed changes in the composition of holothuroid feces under different temperatures. Thus, further experiments measuring also the actual rates of holothuroid organic matter utilization are needed to sustain and deepen our contentions. Nevertheless, our results allow us concluding that holothuroids, like *H. tubulosa*, are confirmed as a potential tool to naturally manipulate quantity and biochemical composition of marine sediments and, prospectically, to mitigate marine benthic eutrophication, even under extreme sea temperature regimes.

## Materials and methods

### ***Sediment sampling***

Sediments were collected in two sites (5–10 m depth; Mediterranean Sea): one located near a mariculture plant in the Gulf of Oristano (Western Sardinia, Mediterranean Sea), characterized by muddy sediments, and one, located in the Gulf of Teulada (Southern Sardinia, Mediterranean Sea), characterized by sandy-mud sediments and nearby meadows of the endemic seagrass *Posidonia oceanica* (Delile, 1813). These two sites were previously ranked as meso-eutrophic (Oristano) and oligo-mesotrophic (Teulada)^19^, based on mean biopolymeric carbon C contents^10,48^. The upper layer (2 cm) of surface sediments from both sites were scraped by scuba divers in December 2020 with 50-mL Falcon-type tubes. Sediments collected from each site were mixed, homogenized, and stored into sterile 250-mL jars at − 20 °C until mesocosms preparation.

### Sea cucumbers’ sampling and holding tanks

Specimens of *H. tubulosa* (mean wet weight 108.8 ± 35.3 g) were collected in the same sites from which sediments were sampled. All holothuroid specimens were kept, under in situ temperature (14 °C) and running seawater, in two 350-L tanks (one per sediment type, each with 1 cm-thick layer of the original sediment) till the initiation of the acclimation phase (see below). The trials were carried out at the experimental aquaculture facility of the University of Cagliari (SW Sardinia, Italy).

### Experimental set-up

Thermally isolated 350-L tanks were filled with seawater and equipped with heaters, thermostats, and thermometers to control and maintain temperature at the desired values. Each of these tanks contained smaller 150-L tanks in which sea cucumbers were acclimated (see below for details) and then starved prior to the feeding and feces production experiments. The large thermally stable 350-L tanks were also used to host the small 6-L tanks used during the feeding and feces collection phases.

Sea cucumbers, before the feeding and feces production phases, were gradually (0.5 °C per day till the chosen temperature) acclimated to 14, 17, 20, 23, 26, 29 °C, with 14 °C representing the minimum temperature faced by *H. tubulosa* specimens in winter^73^ as well as the minimum average sea surface winter temperature in the Mediterranean Sea between 2003 and 2019^77,78^. The temperature of 29 °C was chosen to represent the potentially exacerbated warmest condition, even above that observed in the Mediterranean Sea during several marine heat waves^79–81^.

For both sediment types (n = 2; meso-eutrophic vs. oligo-mesotrophic), 6-L tanks (n = 3) with one sea cucumber each, were prepared per each temperature. During acclimation, sediment and sea cucumbers were maintained at salinity and dissolved oxygen constant levels (36.5 and above 6 mg/L, respectively). During acclimation, ½ of the tank volume was replaced every 3 days, using seawater with a temperature equal to that reached at the day of water exchange. Once all established temperatures were achieved, 3 sea cucumbers per each experimental temperature and sediment type were translocated in thermally preconditioned 150-L tanks and starved without sediments for 72 h (time required to completely empty the sea cucumbers intestine^82^). During starvation, sea cucumbers were placed on a 1-cm mesh net to let feces sinking on the tank bottom and, thus, avoid coprophagia. During the feeding phase, replicate (n = 3) 6-L mesocosms per each experimental temperature and sediment typology were filled with a 1-cm thick layer of original sediment and thermally preconditioned sea water (1:20 v/v). One sea cucumber was then placed in each 6-L tank (gently aerated to avoid water stratification and ensure adequate oxygenation) and left to feed on sediment for 12 h. At the end of the feeding phase, sediments were collected and immediately stored at − 20 °C till the analyses. After the feeding phase, all sea cucumbers were translocated in separate thermally stable, empty (i.e., without sediment) 6-L tanks and feces were collected every 6–8 h for the subsequent 72 h. Feces produced by each specimen were stored in 10-mL PPE tubes at − 20 °C, until analysis.

During the experiment, no specimen died, and, after the experiment, all individuals were relocated at the original sampling site, so to be compliant with the ARRIVE guidelines^83^. The experiment, being carried out with not-cephalopode invertebrate animals, was not subjected to the rules of the EU Directive 2010/63/EU.

### Quantity and biochemical composition of organic matter in sediments and feces

Protein, carbohydrate and lipid contents of sediments and holothuroid feces were determined spectrophotometrically according to the protocols detailed in Danovaro^84^. More in details, protein contents were determined according to Lowry et al.^85^, as modified by Hartree^86^ and Rice^87^, using the Folin‐Ciocalteau reagent in a basic environment and expressed as bovine serum albumin equivalents. The procedure^88^, based on the phenol and concentrated sulfuric acid reaction with saccharides, was used to determine carbohydrates, then expressed as D (+) Glucose equivalents. Lipids, after extraction in chloroform: methanol (1:1, vol:vol^89^, and evaporation in a dry hot bath at 100 °C for 20 min, were determined after the sulfuric acid carbonization procedure^90^ and expressed as tripalmitin equivalents. For each biochemical assay, blanks were obtained using pre‐calcinated (450 °C for 4 h) sediments or feces. All the analyses were performed in triplicate, with about 1 g of sediment or feces per replicate. Protein, carbohydrate, and lipid concentrations were converted into C equivalents using the conversion factors 0.49, 0.40, and 0.75 mgC mg ^-1^, respectively, obtained from the C contents of the respective standard molecules (albumin, glucose and tripalmitin, respectively), and their sum was reported as biopolymeric C (BPC)^91^.

### Effects magnitude

To compare the magnitude of the organic matter enrichment of holothuroids feces at different temperatures, in both meso-eutrophic and oligo-mesotrophic conditions, forest plot representations were drawn based on the ln–response ratio metric calculated as follows:

$${\text{R}}_{{\text{i}}} = {\text{ln}}\;\left( {{\text{F}}_{i} /{\text{S}}_{i} } \right)$$

where, F_i_ and S_i_ are organic matter contents of feces and sediments, respectively, per each specimen at the different experimental temperature (i.e., 14, 17, 20, 23, 26, 29 °C).

### Statistical analyses

Non-parametric permutational analyses of variance (PERMANOVA^92,93^) were performed to test for differences in organic matter quantity and biochemical composition (in terms of protein, carbohydrate, and lipid contents) between sediment and feces across sites and temperatures, followed by post-hoc tests in the case of significant effects (p < 0.05). The design included three fixed orthogonal factors: Site (S; meso-eutrophic vs. oligo-mesotrophic), Matrix (M; sediments vs. feces) and Temperature (T; 14, 17, 20, 23, 26, 29 °C), with n = 3 for the combination of factors. PERMANOVA tests were conducted on Euclidean distance-based resemblance matrices obtained from previously normalized data, using 999 random permutations of the appropriate units and the p-values were obtained after Monte Carlo simulation. PERMANOVA on one response variable using Euclidean distance yields the classical univariate F statistic and can be used, as in this study, to do univariate ANOVA where p values are obtained by permutation^94^, thus avoiding the assumption of normality^95^. Multivariate differences in organic matter biochemical composition (in terms of protein, carbohydrate, lipid) between sediment and feces under different experimental assets (separately for the two trophic status conditions) were visualized with biplots obtained after a canonical analysis of the principal coordinates (CAP)^94^. CAP allows identifying an axis through the multivariate cloud of points that is best at separating a priori groups^95^. All the statistical analyses were performed using the routines included in the PRIMER 6 + software^95^.

## Supplementary Information

Supplementary Information.

## Data Availability

All data generated or analyzed during this study are included in this article and its supplementary information files.
