# Supplementary material for: Sea cucumbers bioturbation potential outcomes on marine benthic trophic status under different temperature regimes
Source: Sci Rep. 2023 Jul 18;13:11558. doi: 10.1038/s41598-023-38543-6 (PMC10354198; doi:10.1038/s41598-023-38543-6)
Supplement: Supplementary file 1 — Supplementary Information. [file 41598_2023_38543_MOESM1_ESM.docx]

Sea cucumbers bioturbation potential outcomes on marine benthic trophic status under different temperature regimes

Claudia Ennas^1+^, Viviana Pasquini^1+^, Hiba Abyaba^1,2^, Pierantonio Addis^1^, Gianluca Sarà^3^, Antonio Pusceddu^1*^

^1^Università degli Studi di Cagliari, Dipartimento di Scienze della Vita e dell’Ambiente, Cagliari 09126, Italy

^2^Scuola Universitaria Superiore IUSS Pavia, Pavia 27100, Italy

^3^Università degli Studi di Palermo, Dipartimento di Scienze della Terra e del Mare, Palermo 90123, Italy

**Supplementary Information**

**Table S1.** Protein, carbohydrate, lipid, and biopolymeric C contents (mean ± standard error, n=3) in sediments and feces at the six temperatures (14, 17, 20, 23, 26, 29°C) in meso-eutrophic (M-E) and oligo-mesotrophic (O-M) conditions.

| **Matrix** | **Trophic Status** | **T (°C)** | **Protein**  **mg g^-1^** | **Carbohydrates**  **mg g^-1^** | **Lipid**  **mg g^-1^** | **Biopolymeric C**  **mgC g^-1^** |
| --- | --- | --- | --- | --- | --- | --- |
| **Sediment** | **M-E** | **14** | 2.30±0.49 | 2.08±0.05 | 0.27±0.06 | 2.16±0.28 |
|  |  | **17** | 1.72±0.30 | 1.04±0.23 | 0.25±0.01 | 1.44±0.24 |
|  |  | **20** | 1.69±0.26 | 0.95±0.06 | 0.11±0.03 | 1.29±0.17 |
|  |  | **23** | 1.48±0.16 | 0.29±0.05 | 0.13±0.03 | 0.94±0.10 |
|  |  | **26** | 2.03±0.25 | 1.06±0.19 | 0.11±0.03 | 1.50±0.20 |
|  |  | **29** | 2.39±0.34 | 1.27±0.12 | 0.54±0.12 | 2.08±0.08 |
|  | **O-M** | **14** | 0.10±0.01 | 0.20±0.01 | 0.01±0.00 | 0.14±0.01 |
|  |  | **17** | 0.10±0.03 | 0.11±0.02 | 0.04±0.03 | 0.13±0.03 |
|  |  | **20** | 0.10±0.01 | 0.17±0.02 | 0.01±0.00 | 0.13±0.01 |
|  |  | **23** | 0.16±0.01 | 0.16±0.03 | 0.01±0.00 | 0.15±0.01 |
|  |  | **26** | 0.16±0.01 | 0.13±0.01 | 0.01±0.00 | 0.14±0.01 |
|  |  | **29** | 0.15±0.02 | 0.12±0.01 | 0.01±0.00 | 0.13±0.01 |
| **Feces** | **M-E** | **14** | 3.36±0.30 | 3.04±0.40 | 0.33±0.06 | 3.11±0.31 |
|  |  | **17** | 2.82±0.92 | 2.88±0.45 | 0.52±0.11 | 2.93±0.42 |
|  |  | **20** | 25.01±5.05 | 3.76±0.13 | 4.65±1.08 | 17.25±3.26 |
|  |  | **23** | 2.53±0.85 | 7.45±0.98 | 0.45±0.11 | 4.56±0.83 |
|  |  | **26** | 6.44±1.02 | 5.64±1.53 | 0.85±0.23 | 6.05±1.19 |
|  |  | **29** | 3.82±0.96 | 5.25±1.68 | 0.61±0.12 | 4.43±1.23 |
|  | **O-M** | **14** | 0.34±0.06 | 0.31±0.03 | 0.13±0.02 | 0.39±0.04 |
|  |  | **17** | 0.15±0.06 | 0.23±0.00 | 0.09±0.02 | 0.23±0.02 |
|  |  | **20** | 0.40±0.11 | 0.50±0.11 | 0.14±0.03 | 0.50±0.01 |
|  |  | **23** | 0.80±0.24 | 0.68±0.18 | 0.15±0.03 | 0.78±0.19 |
|  |  | **26** | 0.39±0.02 | 0.44±0.11 | 0.14±0.03 | 0.47±0.06 |
|  |  | **29** | 0.58±0.06 | 0.53±0.15 | 0.06±0.02 | 0.54±0.10 |

Figure S1. Variations in the concentration of proteins (A-D), carbohydrates (B-E), and lipids (C-F) in OM in sediments and feces at the six temperatures (14, 17, 20, 23, 26, 29°C) displayed separately for the two meso-eutrophic and oligo-mesotrophic conditions. The error bars indicate the standard error (n = 3).
